# Herd immunity and a vaccination game: An experimental study

**DOI:** 10.1371/journal.pone.0232652

**Published:** 2020-05-14

**Authors:** Wooyoung Lim, Pengfei Zhang

**Affiliations:** 1 Department of Economics, Hong Kong University of Science and Technology, Hong Kong, Hong Kong; 2 Department of Economics, Cornell University, Ithaca, New York, United States of America; Middlesex University, UNITED KINGDOM

## Abstract

Would the affected communities voluntarily obtain herd immunity if a cure for COVID-19 was available? This paper experimentally investigates people’s vaccination choices in the context of a nonlinear public good game. A “vaccination game” is defined in which costly commitments (vaccination) are required of a fraction of the population to reach the critical level needed for herd immunity, without which defectors are punished by the natural contagion of epidemics. Our experimental implementation of a vaccination game in a controlled laboratory setting reveals that endogenous epidemic punishment is a credible threat, resulting in voluntary vaccination to obtain herd immunity, for which the orthodox principle of positive externalities fails to account. The concave nature of the infection probability plays a key role in facilitating the elimination of an epidemic.

## Introduction

Globally, infectious diseases are responsible for one-quarter of all deaths each year; even when infections do not kill, they reduce the quality of life of the hundreds of millions of people affected [1]. Recent outbreaks, such as SARS in 2003, Ebola in 2014, MERS in 2015 and currently COVID-19, have forced citizens around the world to confront and reconsider the high social and economic costs of epidemic control. The problem of epidemic control is of fundamental economic importance because disease and infection are irrefutably associated with uncertainty and externalities, which have long been central concepts in economics. According to a WHO report [2], behavioral changes at the community level are key to the prevention and elimination of epidemics, especially in low- and middle-income countries (see also [3] for an excellent recent review on how social and behavioral science can inform behavioral changes to combat COVID-19). Understanding people’s choices for epidemic control thus has significant policy implications.

Our work builds critically on the nonlinear nature of vaccination externality, the foundation of which lies in a class of well-established epidemiological models-the susceptible-infected-recovered (SIR) framework. The SIR model, introduced by [4], describes the transmission of infectious disease through individuals in a society with a fixed population that consists of three compartments: susceptible, infected, and recovered. [5] extended the model to incorporate vaccination with fixed timing and revealed that the long-run probability of infection exhibits an interesting property: it decreases concavely as vaccination coverage increases and eventually vanishes when a critical mass of the population obtains vaccinations. The minimum fraction of a population that must be vaccinated to prevent an outbreak is called “herd immunity.” The public good nature of herd immunity has a behavioral implication that free riders exist, as not all people need to be vaccinated. The nonlinear public goods game, in which forward-looking individuals make vaccination choices to optimize their health assets to guard against epidemics, with the steady-state probability of infection derived from the dynamic epidemic contagion model of [5], is in our formulation the “vaccination game.”

We experimentally explore how the nonlinear nature of this prevention externality affects the individuals’ vaccination choice problem. In our laboratory vaccination game there are eight individuals, each of whom independently and simultaneously decides whether to be vaccinated. Each subject faces a trade-off between the nonlinear probabilistic benefit of vaccination and the deterministic cost of vaccination, although vaccination behavior benefits society as a whole. For each individual, the benefit of being vaccinated monotonically and nonlinearly decreases with the number of other people being vaccinated. We explore various treatments that differ in the relative benefit of vaccination and the characteristics of the disease (the reproduction ratio).

Our experimental results of the controlled laboratory setting provide strong support for the major theoretical predictions. The experimental data help us reject the predictions from pure-strategy equilibria, and show that there is a strategic advantage from being unpredictable in immunization, which leads subjects to randomize in their vaccination choice. The (symmetric) mixed-strategy equilibrium predicts that an increase in the relative benefit of vaccination results in unambiguously higher vaccination coverage and, consequently, a higher likelihood of herd immunity. The mean vaccination coverage and the likelihood of herd immunity reported in our experimental data confirm that endogenous epidemic punishment is indeed a credible threat for voluntary vaccination, indicating that herd immunity can be achieved through voluntary, private vaccination.

The concave nature of the infection probability plays a key role in facilitating the elimination of an epidemic. To elaborate this point, we compare the concave environment with a benchmark, linearized environment where a hypothetical linear approximation of the infection probability is adopted. Our theoretical analysis suggests that given any cost of vaccination and any type of disease, the equilibrium vaccination coverage under the concave infection probability always first-order stochastically dominates that under the linear infection probability. This happens precisely because under the concave infection probability, the vaccine uptake likelihood of any individual grows at a *higher marginal rate* with a decrease in vaccination cost. Our experimental data confirm this observation. Our finding suggests that policies targeting at a lower vaccination cost (e.g. fiscal subsidy, public health campaign and R&D) would be effective to achieve herd immunity via voluntary vaccination.

## Methods

### Description of the game

In our vaccination game, forward-looking individuals make vaccination choices to optimize their health assets to guard against epidemics. Each decision maker thus faces a trade-off between the probabilistic benefit of vaccination and the deterministic cost of vaccination, although vaccination behavior benefits society as a whole. [5] show that the probability of infection decreases concavely as vaccination coverage rises and eventually vanishes when a critical mass of the population obtains vaccinations. The concavity of epidemic externality has a natural interpretation from positive network externality (see [6]; [7]). The minimum fraction of a population that must be vaccinated to prevent an outbreak is called herd immunity. The public good nature of herd immunity gives rise to a strategic vaccination problem since individual vaccination choice affects others’ payoff through the endogenously determined probability of infection.

Consider a society with a finite set of players N≔{1,2,…,n} whose preferences satisfy the Von Neumann-Morgenstern axioms such that they maximize expected utility. Each player i∈N simultaneously and independently makes his vaccination choice *b*_*i*_ in $B_{i}≔\left\{vc,nv\right\}$ where *vc* and *nv* refer to vaccination and no vaccination choices, respectively. For any player *i*, let *b*_−*i*_ = (*b*_1_, …*b*_*i*−1_, *b*_*i*+1_, …, *b*_*n*_) denote the pure-strategy profile of other players. For a given pure-strategy profile *b* = (*b*_1_, …, *b*_*n*_) = (*b*_*i*_, *b*_−*i*_), let $V\left(b\right)=\left\{i\in N|b_{i}=vc\right\}$ denote the set of vaccinated players. The proportional vaccine coverage of the population *P* is endogenously determined as follows:

P(b)=|V(b)|/n,

where |*V*(***b***)| = *ν* denotes the cardinality of the set *V*(***b***).

Define *P*_*crit*_ as the vaccination coverage needed for herd immunity after which the entire population will be safe from an epidemic. Based on the well-established SIR model in mathematical epidemiology (refer to, e.g., [4]; [8]) [5] shows that

$$\begin{array}{c} P_{crit}=1-\frac{1}{R_{0}}, \end{array}$$ (1)

where *R*_0_ is the basic reproduction ratio of the disease. Note that *R*_0_ > 1 for any epidemic (see [9] for a detailed discussion). In the subsequent analysis, we use *R*_0_ as a general characteristic that differentiates epidemics.

Let *ν*_*crit*_ denote the smallest integer value above *P*_*crit*_ *n*. For a given *P*, let *π*_*P*_ denote the long-run infection probability. Taken from the result in [5], we assume that $\pi_{P}=1-\frac{1}{R_{0}\left(1-P\right)}$ is strictly decreasing and concave in *P* for any *P* < *P*_*crit*_, and *π*_*P*_ = 0 for any *P* ≥ *P*_*crit*_.

For each player *i*, there are three possible health statuses *ex post*: susceptible (S), infected (I), and recovered (R). For any health status *θ* ∈ {*S*, *I*, *R*}, *u*(*θ*) denotes the instantaneous utility from the state *θ*. Assume that

$$\begin{array}{c} u(S)=u(R)>u(I), \end{array}$$ (2)

such that a player values the uninfected status qualitatively more than the infected status. Define the utility cost of infection as *L* = *u*(*R*) − *u*(*I*).

For any player *i*, the expected utility of not vaccinating given a fixed strategy profile of other players *b*_−*i*_ (and thus for a fixed vaccine coverage *P*) is as follows:

$$\begin{array}{c} U_{i}\left(nv,b_{-i}\right)=\{\begin{array}{cc} \frac{1}{\mu }u\left(R\right) & if\;P\left(nv,b_{-i}\right)\geq P_{crit}\\ \frac{1}{\mu }u\left(S\right)-\pi_{P(nv,b_{-i})}\cdot d_{R_{0}}\cdot L & if\;P\left(nv,b_{-i}\right)<P_{crit}, \end{array} \end{array}$$

where 1/*μ* is the expected life expectancy, $d_{R_{0}}>0$ is the duration of the infection uniquely determined by *R*_0_, the basic reproduction ratio of the disease. $d_{R_{0}}^{\prime }\geq 0$ because a larger *R*_0_ implies a lower possibility of recovery.

For any player *i*, the expected utility of vaccination for any *b*_−*i*_ is

$$\begin{array}{c} U_{i}\left(vc,b_{-i}\right)=U_{i}\left(vc\right)=\frac{1}{\mu }u\left(R\right)-C, \end{array}$$

where *C* is the utility cost of vaccination that may reflect a combination of cash costs, psychological costs and possible side effects. All of these are common knowledge.

Let $r=\frac{L}{C}\in \left(\frac{R_{0}}{\left(R_{0}-1\right)d_{R_{0}}},+\infty \right)$ be the relative benefit of vaccination. We call the simultaneous-move game defined by the tuple $\left\{N,{\left\{B_{i}\right\}}_{i\in N},{\left\{U_{i}\right\}}_{i\in N}\right\}$ the vaccination game G.

### Equilibrium of the game

In this game, there is a strategic advantage from being unpredictable in immunization, which could lead individuals to randomize in taking vaccination. Consider why an individual would use a mixed strategy for the immunization choice in the simplest three-player setting when one of them has already been infected and the vaccination of only one susceptible individual suffices to obtain herd immunity in the society. If the other susceptible individual has not vaccinated for certain, an individual should protect himself/herself from infection unless the vaccination cost is unreasonably high; however, if the other individual is certain to vaccinate, an individual would choose to free ride. Therefore, there is a strategic advantage of being unpredictable.

The full characterization of Nash equilibrium outcomes is presented in S1 Appendix. In the appendix, we show that there exists three classes of equilibria: a spectrum of pure-strategy equilibria, a spectrum of partially mixing equilibria, and a unique totally mixing equilibrium where every player uses a mixed strategy. The asymmetry of the first two classes of equilibria is undesirable because they all arbitrarily require identical players to choose different strategies in a precisely coordinated manner. See [10] for a detailed discussion of this coordination issue. For the same reason, [11] focus on the symmetric mixed-strategy equilibrium in their experimental investigation of the volunteer’s dilemma. On the other hand, the vaccination game has no asymmetric totally mixed-strategy Nash equilibrium. In the subsequent analysis, we thus focus on the unique totally mixed-strategy equilibrium. We shall see later if any of the asymmetric equilibria could explain the observed laboratory behavior well.

**Fig 1:**
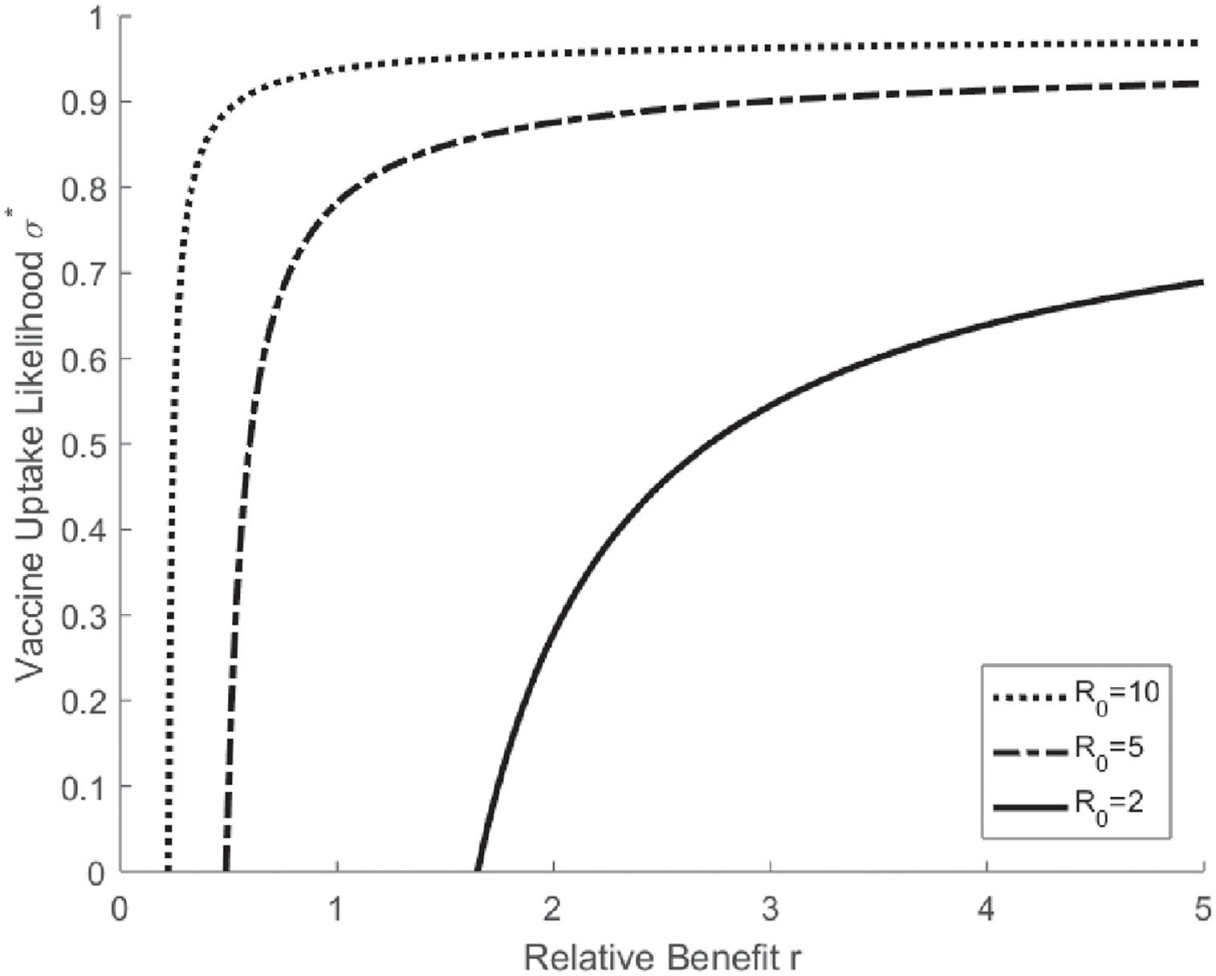
Mixed strategy and herd immunity.

In this equilibrium, an increase in the relative benefit of vaccination results in an unambiguously higher vaccination coverage and, consequently, a higher likelihood of herd immunity. The proofs of these propositions can also be found in S1 Appendix. Assuming a linear form of duration $d_{R_{0}}=0.5R_{0}$ and *n* = 200, Fig 1 plots the equilibrium likelihood of vaccine uptake σ* as a function of *r* for different types of diseases. It illustrates that the individual vaccination uptake likelihood σ*(*r*, *R*_0_) is strictly increasing in *r* and converges to 1 as *r* goes to infinity. That is, if the cost of vaccination is far less than the loss of contracting the disease, people are almost certain to opt for vaccination. In the extreme case of a fatality, the substantially high relative benefit *r* is not only effective in enforcing voluntary vaccinations but also deters free riders who leave their fates to others.

### Nonlinearity plays a key role

The concavity of the infection probability plays a key role in facilitating the elimination of an epidemic. To elaborate this point, we compare the concave environment with a linearized environment where a hypothetical linear approximation of the infection probability is adopted. Our theory suggests that given any cost of vaccination and any type of disease, the equilibrium vaccination coverage under the concave infection always first-order stochastically dominates that under the linear infection. This happens precisely because under the concave infection, the vaccine uptake likelihood of any individual grows, at a *higher marginal rate*, with a decrease in vaccination cost.

**Fig 2:**
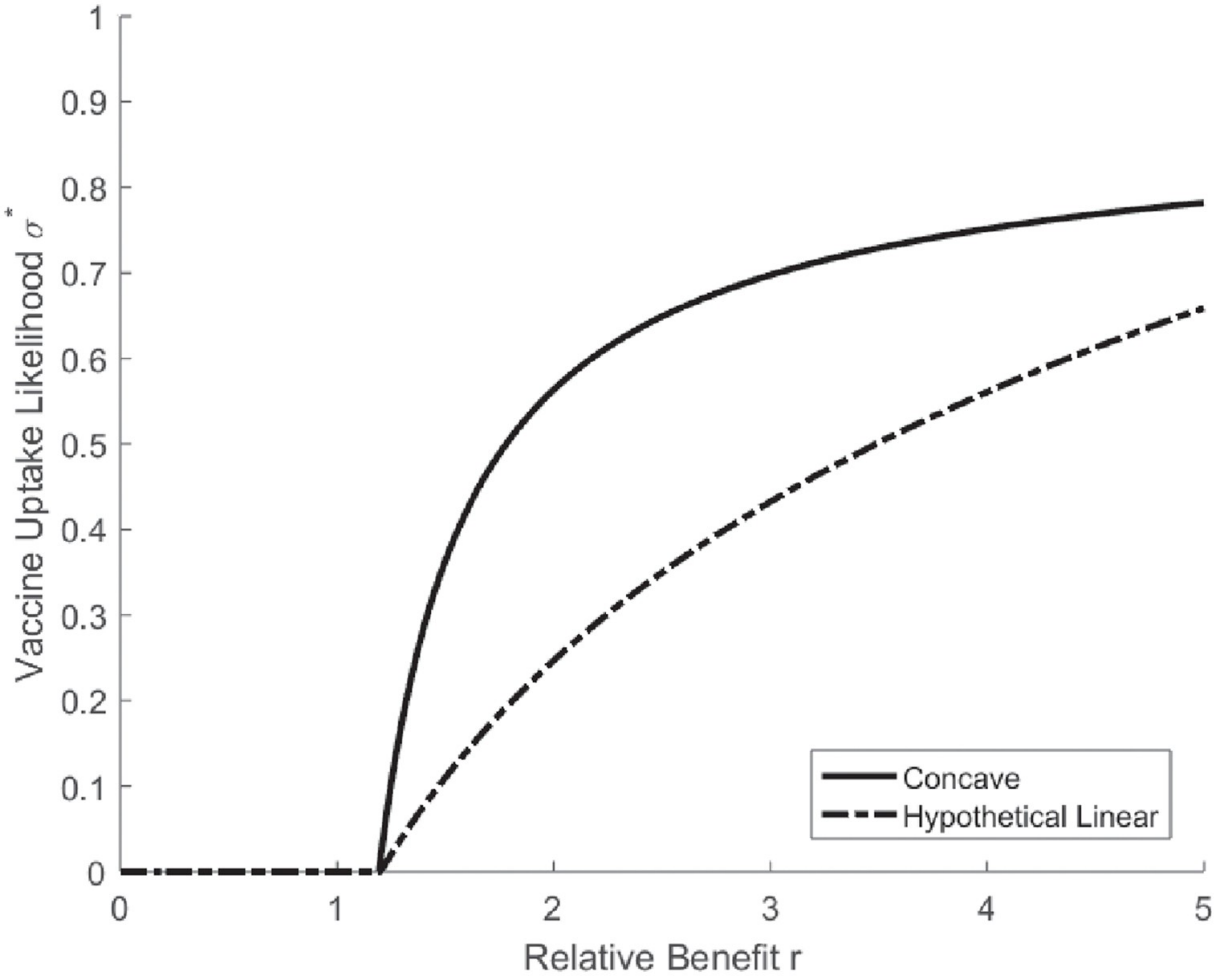
Elimination thresholds in concave vs. linear environment.

The concavity of *π*_*P*_ implies that the vaccination uptake likelihood σ* from the mixed-strategy Nash equilibrium is strictly increasing and *concave* in the relative benefit *r*, as illustrated in Fig 2. Fig 2 also presents the vaccination uptake likelihood based on $\pi_{P}^{L}$, a counterfactual, linear approximation of *π*_*P*_. In this hypothetical “linearized” environment, the vaccination uptake likelihood becomes less concave in *r*. It is thus more difficult to achieve herd immunity via voluntary vaccination. In our experiment, we design treatments to test this hypothesis.

### Experimental design

We now present our experimental design. Except for the work by [12], no experimental study has directly investigated how the benefit and cost of vaccination influence free-riding behavior in individuals’ vaccination decisions. To the best of our knowledge, we are the first to experimentally explore how the nonlinear nature of the positive prevention externality affects people’s free-riding behavior in the context of a vaccination choice problem.

### Treatments and hypotheses

Our experimental implementation considers the following reduced form of the vaccination game. There are eight individuals in a society (i.e., *n* = 8), and they are ex ante identical. Each individual is initially endowed with *u*(*R*)/*μ* = 80. It is assumed that the duration of infection takes the functional form $d_{R_{0}}=0.8R_{0}$ and the utility cost of vaccination *C* = 5. That is, with herd immunity, all free riders receive 80, while people who receive the vaccine receive 75. In the absence of herd immunity, the probability of infection *π*_*P*_ is endogenously determined by *P*, according to Proposition 1. For example, when the utility cost of infection *L* = 25, the basic reproduction ratio is *R*_0_ = 4, and the vaccination coverage is *P* = 1/2, so *π*_*P*_ = 1/2; i.e., the payoff for a nonvaccinator is equally likely to be 80 and 0 (= 80 − 0.8 × 4 × 25).

The major treatment variables correspond to the relative benefit of vaccination (*r*: relative benefit) and the basic reproduction ratio of the epidemic (*R*_0_: reproduction ratio). We choose parameter specifications with *r* = 1 or 5 and *R*_0_ = 2 or 4 to create a qualitative difference in the vaccination coverage predicted by the symmetric mixed-strategy Nash equilibrium across treatments. Table 1 presents the four treatments, each of which involves a unique combination of the relative benefit and basic reproduction ratio as well as the equilibrium predictions. We also have one more treatment (Treatment 3L) that incorporates the hypothetical linearized environment discussed in the previous section into the set of parameters chosen for Treatment 3.

**Table 1:** Experimental treatments and theoretical predictions.

Treatment	Parameter Specification		Theoretical Predictions			
	Relative Benefit	Reproduction Ratio	Herd Immunity	Mixed Strategy	Expected Number of Vaccinated People	Likelihood of Herd Immunity
	*r*	*R*_0_	*nP*_*crit*_	σ*	nE[P]	Pr(*P* ≥ *P*_*crit*_)
1	1	2	4	0	0	0%
2	5	2	4	0.52	4.16	67.95%
3	1	4	6	0.68	5.44	50.13%
4	5	4	6	0.89	7.12	95.13%

A unique feature of Treatment 1 is that the reproduction ratio *R*_0_ is large relative to the relative benefit *r*, indicating that the expected gain from vaccination is dominated by the expected cost. As a result, no vaccination is predicted. With the three other treatments, however, the individual probability of vaccination is positive in the mixed-strategy equilibrium, and the resulting vaccination coverage for the society reaches the critical level needed for herd immunity in more than 50% of cases. Thus, we have our first hypothesis as follows.

**Hypothesis 1**.

*(a) In Treatment 1, the individual probability of vaccination is not significantly different from 0, and as a result, the likelihood of herd immunity is not significantly different from 0*.
*(b) In Treatments 2, 3 and 4, the individual probability of vaccination is significantly higher than 0*.
*(c) In Treatments 2, 3 and 4, the likelihood of herd immunity is significantly higher than 0*.

We next consider whether the endogenous epidemic punishment is effective. Given the reproduction ratio *R*_0_, the higher relative benefit *r* in Treatment 2 (Treatment 4) than in Treatment 1 (Treatment 3) makes it more likely that an individual vaccinates, and thus, the society achieves herd immunity in Treatment 2 (Treatment 4) than in Treatment 1 (Treatment 3).

**Hypothesis 2** *Given a fixed reproduction ratio R*_0_,

*(a) the individual likelihood of vaccination is higher in Treatment 2 than in Treatment 1 and higher in Treatment 4 than in Treatment 3*.
*(b) the likelihood of herd immunity is higher in Treatment 2 than in Treatment 1 and higher in Treatment 4 than in Treatment 3*.

We investigate the effect of the reproduction ratio on individuals’ vaccination choices. Given the relative benefit *r*, the higher reproduction ratio *R*_0_ in Treatment 3 (Treatment 4) than in Treatment 1 (Treatment 2) makes it more likely for an individual to take vaccination in Treatment 3 (Treatment 4) than in Treatment 1 (Treatment 2).

**Hypothesis 3** *Given a fixed relative benefit r*,

*(a) the individual likelihood of vaccination is higher in Treatment 3 than in Treatment 1 and higher in Treatment 4 than in Treatment 2*.
*(b) the likelihood of herd immunity is higher in Treatment 3 than in Treatment 1 and higher in Treatment 4 than in Treatment 2*.

Notably, the predictions from the asymmetric pure-strategy Nash equilibria are invariant to the changes in the treatment variables. With every treatment, there exists a collection of asymmetric equilibria in which exactly *n*⋅*P*_*crit*_ players are vaccinated, and the remainder are not. As a result, the likelihood of herd immunity is expected to be 100% in all four treatments. Our experimental data are informative and enable us to reject this prediction from the asymmetric equilibria.

### Procedures

The experiments were approved by Human Participants Research Panel, Committee on Research Practices at the Hong Kong University of Science and Technology, and were conducted in English at the Experimental Lab using z-Tree [13]. All 16 sessions for the main treatments were conducted between November and December 2015 and 4 other sessions for the additional non-linear treatment were conducted between February and March 2017. A *between-subjects* design and *random-matching* protocol were used. Four sessions were conducted for each of the four treatments, and each session included twenty-four subjects. Using sessions as independent observation units, we have four observations for each treatment. A total of 384 subjects with no prior experience with our experiment were recruited from the undergraduate and graduate populations of the university and participated in 16 sessions. All participants were recruited via email invitations with an online signup document. When they signed up for the experiments, they were asked to read a written consent form carefully and click a button to grant their consent.

Upon arrival at the laboratory, subjects were instructed to sit at separate computer terminals. Each participant was given a copy of the experiment instructions, which were read aloud and supplemented with slide illustrations. In each session, subjects first participated in one practice round and then in 20 official rounds.

We illustrate the instructions for Treatment 1. The full instructions can be found in S2 Appendix. In each round, a subject was randomly matched with seven other participants to form a group of eight. In each group, the eight members were asked to make decisions that would affect their earnings in the round. The participants were randomly rematched after each round to form new groups.

We asked the subjects to imagine that these eight individuals in a group live in a village. Initially, every individual in the village begins with the same green status. There is a red circle that carries the source of redness, from which subjects want to protect themselves. Each individual independently and simultaneously decides whether to buy the shield. The price of the shield is fixed at 5 experimental currency units (ECU). With the shield, a subject is immune to redness and stays green; without the shield, a subject will either turn red or remain green, depending on how many other individuals in the village have the shield. Table 2 presents the probability of turning red.

**Table 2:** Probability that you turn red.

If you choose “No Shield”	
# of others having the shield	Prob. of turning red
0	50.0%
1	42.9%
2	33.3%
3	20.0%
4	0.00%
5	0.00%
6	0.00%
7	0.00%

The earnings in each round are determined by the ex post status of an individual and by whether he/she buys the shield. If the individual does not buy the shield but his/her status remains green, then he/she earns 80 ECU. If his/her status turns red, resulting in the loss of 8 ECU, his/her earnings are 80 − 8 = 72 ECU. If an individual buys the shield, he/she must pay 5 ECU, and his/her status stays green so that he/she earns 80 − 5 = 75 ECU.

We randomly selected one round to determine the subjects’ payments. A subject was paid the amount of ECU that he/she earned in the selected round at an exchange rate of 10 ECU = 1 HKD. A session lasted for approximately forty-five minutes, and the subjects earned, on average, HK$109 (≈US$14), including a HK$30 show-up fee. Under Hong Kong’s currency board system, the HK dollar is pegged to the US dollar at a rate of 1 USD = 7.8 HKD.

## Results

Table 3 presents a summary of our experimental results aggregated over all of the sessions for each treatment. According to Column (1) of Table 3, the individual frequencies of choosing vaccination were significantly greater than zero with all four treatments (*p* < 0.066, signed rank test). Column (2) of the table reveals that, consistent with the theoretical predictions, the mean vaccination coverage was lower than herd immunity in Treatments 1 and 3 and higher than herd immunity in Treatment 2. Moreover, the mean vaccination coverage was surprisingly close to herd immunity in Treatment 4.

**Table 3:** Summary of experimental results.

Treatment	(1) Vaccination Frequency	(2) Number of Vaccinated People	(3) Likelihood of Herd Immunity
1	0.243	1.944	8.75%
2	0.551	4.408	78.75%
3	0.604	4.832	32.50%
4	0.742	5.936	66.67%

**Fig 3:**
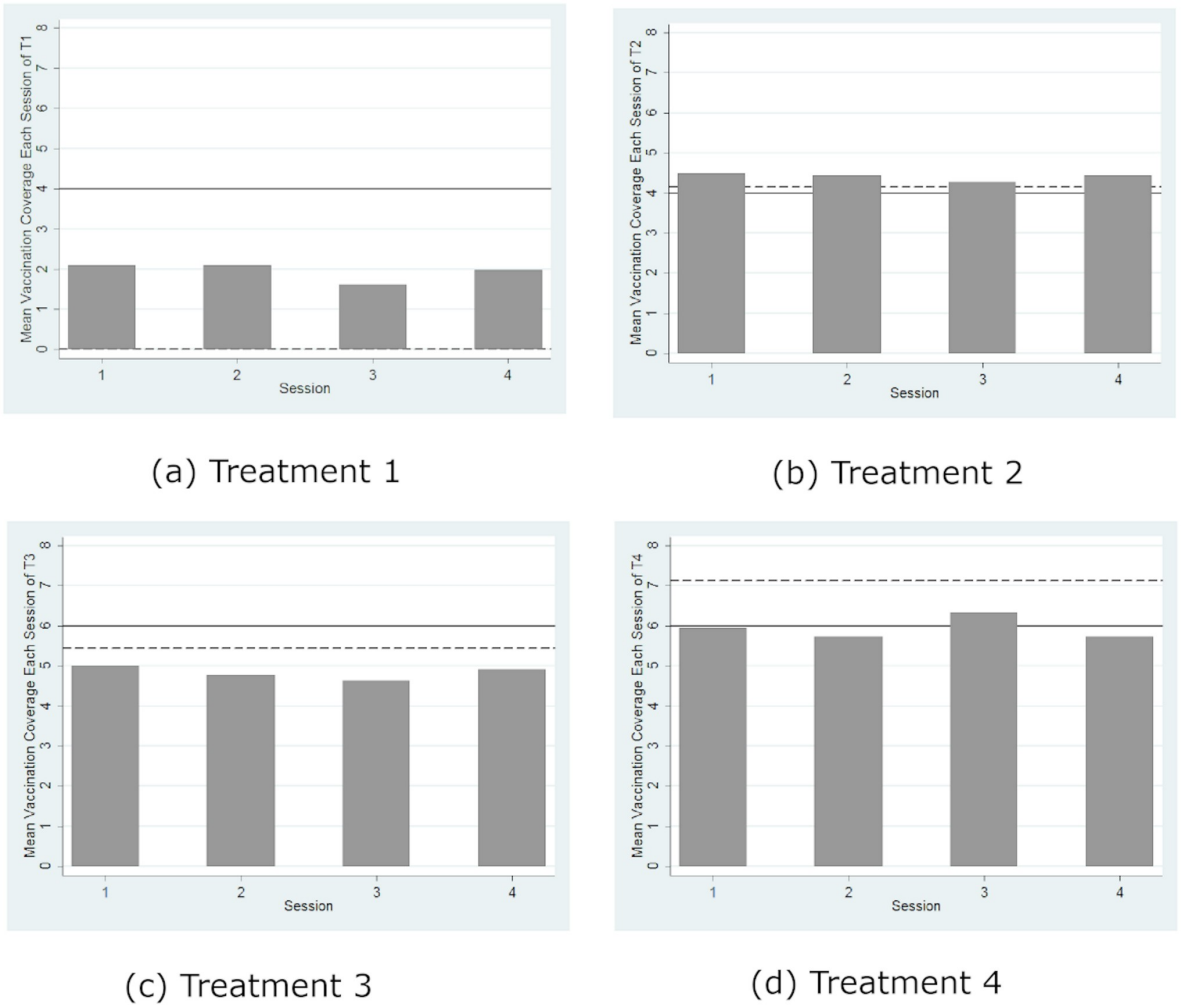
Mean vaccination coverage. The solid line represents herd immunity, and the dashed line represents the Nash prediction.

**Fig 4:**
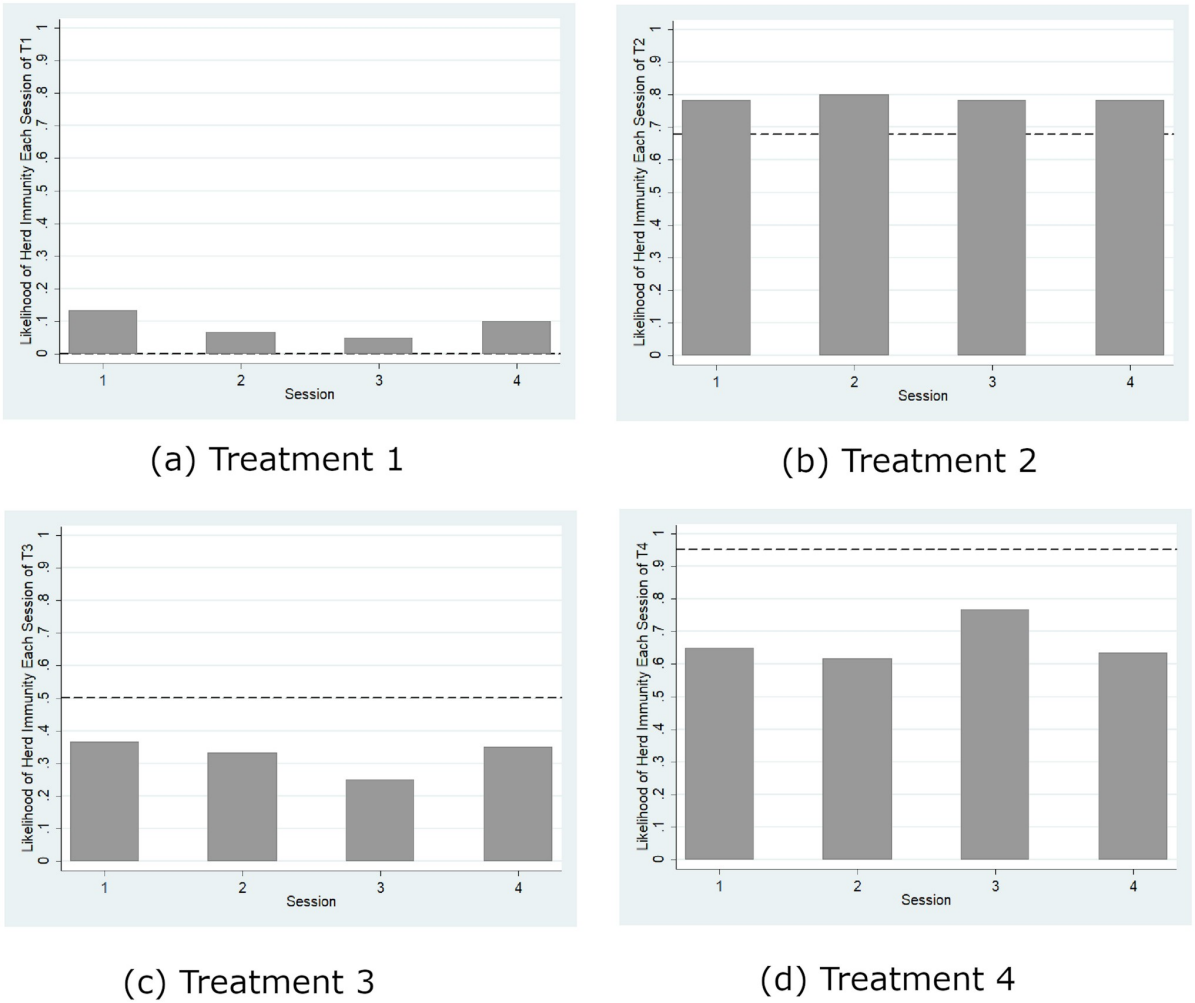
Likelihood of herd immunity. The dashed line represents Nash prediction.

Fig 3 reports the mean vaccination coverage aggregated over all of the rounds for each session of each treatment and compares it with the threshold for herd immunity (represented by solid lines) and the theoretical prediction (represented by dashed lines) from the mixed-strategy Nash equilibrium (Nash prediction hereafter). The mean vaccination coverages in all four sessions in Treatment 1, as shown by Fig 3, were significantly less than the herd immunity level (*p* = 0.066, signed rank test) but significantly greater than the boundary Nash prediction (*p* = 0.066, signed rank test). However, this regularity of a higher actual contribution level than the Nash prediction has been well documented in the standard literature on public good games (e.g., Andreoni, 1995). Similarly, Fig 4 shows that the likelihood of herd immunity aggregated over all of the rounds for each session of each treatment is significantly greater than 0 in all four treatments (*p* < 0.07 for all four cases, signed rank tests). Thus, we confirm Hypotheses 1(b) and 1(c) but reject 1(a). However, inconsistent with the predictions from the pure-strategy equilibria, the likelihood of herd immunity is significantly lower than 100% (*p* < 0.058, all four cases, signed rank test). Thus, we reject the efficient provision of herd immunity predicted by the pure-strategy equilibria.

**Result 1** *In all of the treatments, the individual frequency of choosing vaccination and the likelihood of herd immunity are significantly greater than 0. Moreover, in all of the treatments, the likelihood of herd immunity is significantly less than 100%*.

The endogenous epidemic punishment is a credible threat, inviting voluntary vaccination to obtain the public good of herd immunity. Fig 4 shows that a higher relative benefit led to a significant increase in the likelihood of herd immunity. The Mann-Whitney test confirms that the likelihood of herd immunity was significantly higher in Treatment 2 than in Treatment 1 (*p* = 0.018) and higher in Treatment 4 than in Treatment 3 (*p* = 0.02). We also observed that a higher reproduction ratio led to a significant increase in the likelihood of herd immunity. Again, the nonparametric Mann-Whitney test confirmed that the likelihood of herd immunity was significantly higher in Treatment 3 than in Treatment 1 (*p* = 0.02) and higher in Treatment 4 than in Treatment 2 (*p* = 0.018), thus confirming our Hypotheses 2 and 3.

Fig 8(a) reported in S1 Fig plots the group-level time-trend data for the likelihood of herd immunity. The high volatility observed in Fig 8(a) stems from herd immunity generating a binary outcome, and the social cost was tightly associated with the realization of this outcome. Consistent with the Nash prediction, groups rarely contributed sufficiently to achieve herd immunity in Treatment 1, in sharp contrast to the high frequencies of herd immunity achieved in Treatments 2 and 4. The likelihood of herd immunity was substantially higher than 0 in Treatment 3 (32.5%), also consistent with the theoretical prediction. We have the following results.

**Result 2** *Given a fixed reproduction ratio R*_0_, *the likelihood of herd immunity was higher in Treatment 2 than in Treatment 1 and higher in Treatment 4 than in Treatment 3. Given a fixed relative benefit r*, *the likelihood of herd immunity was higher in Treatment 3 than in Treatment 1 and higher in Treatment 4 than in Treatment 2*.

### Role of concavity: Experimental evidence

In this section, we provide experimental evidence for the role of concavity in enhancing the individual vaccination uptake likelihood and thus the likelihood of herd immunity. To investigate this issue, we considered a new, counterfactual treatment, Treatment 3L, a variant of Treatment 3 in which the probability of infection is linearized. Recall that, in Treatment 3, the long-run probability of infection was nonlinear, as $\pi_{P}=1-\frac{1}{4(1-P)}$. In Treatment 3L, we linearized it to be $\pi_{P}^{L}=\frac{3}{4}-P$. Note that $\pi_{P}^{L}$ is chosen such that $\pi_{P}=\pi_{P}^{L}$ at *P* = 0 and at *P* = *P*_*crit*_. There is no other difference between Treatment 3 and Treatment 3L. Our selection of Treatment 3 for linearizing the environment is guided by the magnitude of change in the individual vaccination uptake likelihood from the linearized *π*_*P*_ being most salient in Treatment 3.

The theoretical prediction confirms our intuition and reveals that the linearized probability of infection decreases the equilibrium vaccination uptake likelihood σ* and the likelihood of herd immunity *Pr*(*P* ≥ *P*_*crit*_). Table 4 presents the parameter choices for Treatment 3L and the theoretical predictions, compared to those of Treatment 3.

**Table 4:** Treatment 3L and its theoretical predictions.

Treatment	Parameter Specification			Theoretical Predictions			
	Relative Benefit	Reproduction Ratio	Long-run Infection Prob.	Herd Immunity	Mixed Strategy	Expected Number of Vaccinated People	Likelihood of Herd Immunity
	*r*	*R*_0_	*π*_*P*_	*nP*_*crit*_	σ*	nE[P]	Pr(*P* ≥ *P*_*crit*_)
3	1	4	$1-\frac{1}{4(1-P)}$	6	0.68	5.44	50.13%
3L	1	4	$\frac{3}{4}-P$	6	0.50	4.00	14.45%

We conducted four sessions of Treatment 3L, and each session had 24 subjects. The same experimental procedure was used. A session lasted for approximately 45 minutes, and subjects earned, on average, HK$105.6 (≈US$13.5), including a HK$30 show-up fee.

**Fig 5:**
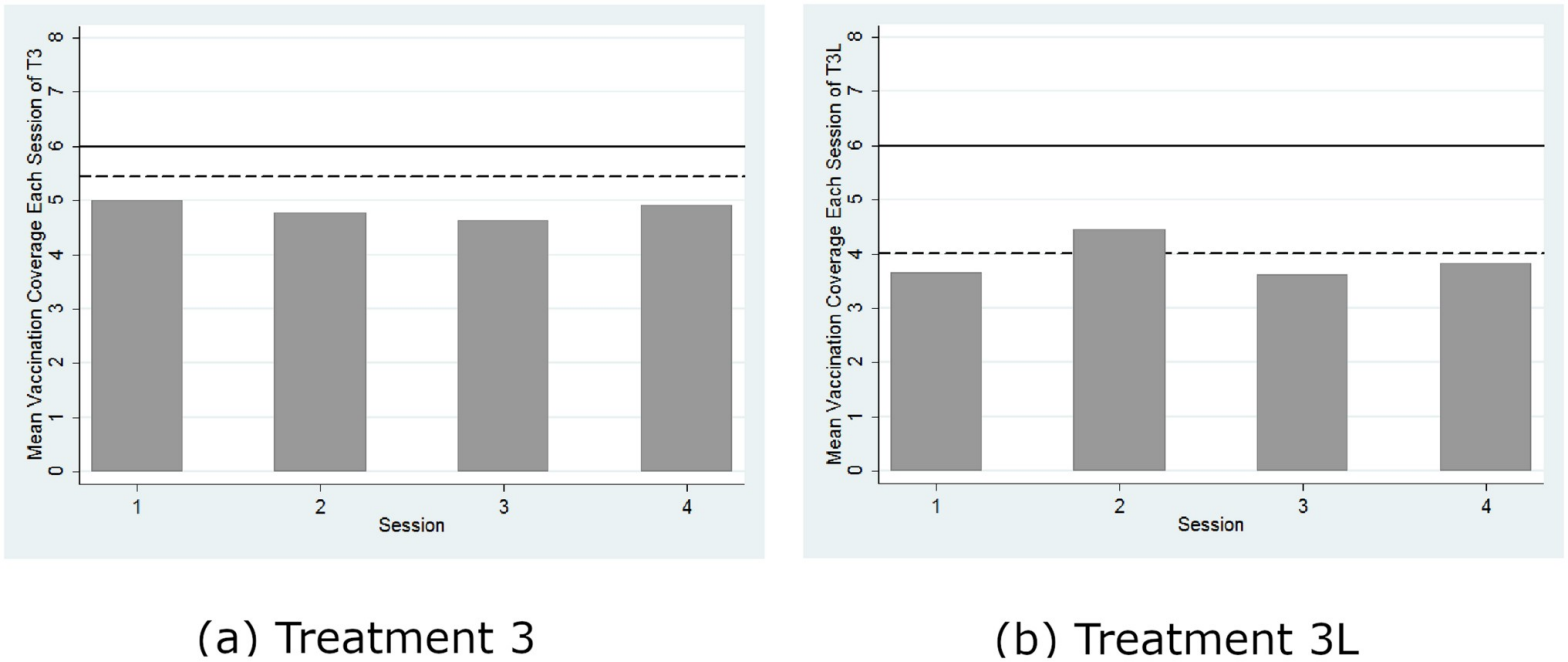
Mean vaccination coverage. The solid line represents herd immunity, and the dashed line represents the Nash prediction.

Fig 5 reports the mean vaccination coverage from Treatment 3L. Confirming the theoretical prediction that the concavity of the long-run probability of infection facilitates individual vaccination uptake likelihood, the Mann-Whitney test revealed that the mean vaccination coverage was significantly higher in Treatment 3 than in Treatment 3L (*p* = 0.02).

**Fig 6:**
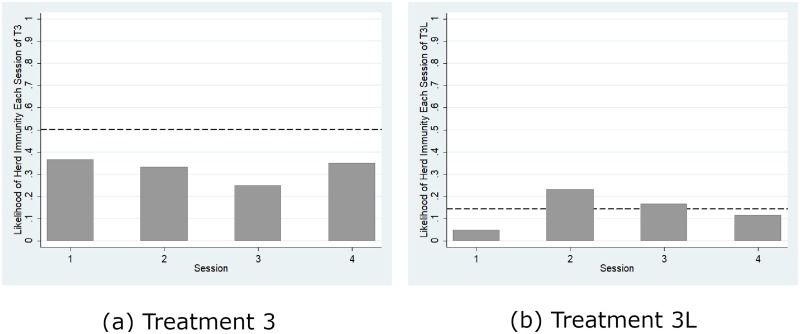
Likelihood of herd immunity. The dashed line represents the Nash prediction.

Fig 6 shows that Treatment 3L is dominated by Treatment 3 with respect to the likelihood of herd immunity. Confirming the theoretical prediction that the concavity of the long-run probability of infection increases the likelihood of herd immunity, the Mann-Whitney test revealed that the likelihood of herd immunity was significantly higher in Treatment 3 than in Treatment 3L (*p* = 0.02). Similarly, Fig 8(c), presented in S2 Fig, shows the same dominance relationship between Treatments 3 and 3L. These results are summarized in the following proposition.

**Result 3** *The individual frequency of choosing vaccination was significantly higher in Treatment 3 than in Treatment 3L. Similarly, the mean likelihood of herd immunity is significantly higher in Treatment 3 than in Treatment 3L*.

## Conclusion

In this paper, we consider a laboratory vaccination game where subjects in a group of eight independently and simultaneously make immunization decisions. We obtain a novel result in the presence of nonlinear prevention externality: for each type of disease, there is a range of relative benefit of vaccination such that epidemics can be eliminated through voluntary vaccination. In addition, our experimental design allows us to separately identify the behavioral response to the positive externality of vaccination (apart from the negative externality of infection).

Policies regarding epidemic control can be better informed by considering individuals’ strategic vaccination choices. Our findings are vital in predicting whether voluntary vaccinations can be an effective mechanism to achieve the social optimum. From the social planner’s perspective, it implies that voluntary vaccination can approximate elimination of any disease if the relative benefit of vaccination is large enough, i.e., when the individual benefit from vaccination is sufficiently larger than the individual’s utility cost of vaccination. A mandatory vaccination program is *not* necessary to achieve the social optimum, especially when the network externality is strong in the society and the nature of externality is concave. The social planner’s well-defined policy goal should be to increase the relative benefit. Several policy tools appears natural to achieve this goal, such as a subsidy to decrease the cash cost of vaccination, a nationwide public vaccination campaign to lower the psychological cost of vaccination, and financial aid to encourage R&D activities to minimize the side-effects of vaccines. Policy prescriptions, on the other hand, should also be interpreted with caution as in uncontrolled settings, many factors including framing, social norm and even emotion, can have salient effects on preventive behavior ([3]).

Our findings contribute to the emerging literature on strategic vaccination ([14], [15], [16]). This literature relaxes the assumption that the risk of infection is invariant to the vaccination coverage and pays particular attention to the strategic interplay of susceptible individuals. [14] showed the efficacy of the vaccine can switch the game from strategic complementarity to subsitutability. [16] considered how the prevalence elastic strategy in a two-player dynamic game of vaccination can yield inefficiently low vaccination uptake. A prevalence elastic strategy is an action plan that is contingent on how many people are infectious over the course of an epidemic. It is usually employed in models featuring flexible timing of vaccination, where feedback about the epidemic is available. As noted by [17], the prevention externality, wherein the vaccination choice of an individual may reduce the likelihood that others are immunized, should be separated from the infection externality, as one’s own infection can increase the likelihood that others are infected. [15] focused on how group structure, in particular, homophily and assortative matching, affects immunization behavior. We advance this literature by showing how a nonlinear externality affects vaccination decisions and demonstrating the key role of concavity in facilitating the elimination of epidemic by voluntary vaccination.

In the economics literature, nonlinear public good games have been studied primarily to test the robustness of the typical over-contribution result observed in the standard linear public good games. Several papers, such as [18], [19] and [20], show that the over-contribution results are persistent in the nonlinear settings with interior Nash equilibria. [21] explore how robust the over-contribution results are to changes in the information describing the payoff structure. They show that, although experimental subjects continue to allocate more resources than the Nash prediction, providing participants with detailed descriptions of the declining marginal benefit to the public good leads to a significant decrease in the provision of public goods. In a recent contribution, [22] study behavior in linear and nonlinear social dilemma games with costly punishment opportunities and demonstrate that the impact of punishment is weaker and takes longer to be effective in a nonlinear environment.

Nonlinear public good games have been also investigated in the context of common pool resources (e.g., [23]; [24]) and of the volunteer’s dilemma (e.g., [25]; [26]; [11]). In the volunteer’s dilemma initiated by [25], only a single volunteer’s costly commitment to provide a public benefit is required, and a symmetric equilibrium usually involves mixed strategies. In fact, [26] classify the volunteer’s dilemma as a special case of a threshold public good, where the critical level requires a fraction of the population. [27] further generalizes threshold public good games to a class of social dilemmas parametrized by the marginal return of cooperation, and studies an experimental setting where the benefit of cooperation increases linearly up to the threshold and then plateaus. While their focus is on group size effect, we instead fix the group size and vary the curvature and the threshold. Our experiment provides novel and complementary evidence that the convexity of the benefit function further improves contribution over its linear counterpart. Moreover, a standard provision point discrete public good game (see [28]) usually exhibits multiple equilibria in pure strategies, and mixing is usually not a concern. To the best of our knowledge, the vaccination game is the first to consider to mixed-strategy Nash equilibria in nonlinear threshold public good games.

For future work, it would be interesting to explicitly consider the timing of vaccination in the experimental setting. In some instances people receive regular feedback about the number of infected people and the proportion of vaccinated individuals in society, such that from time to time, people update their beliefs before making their vaccination choices. In this case, the strategic optimal timing of vaccination is important. It is thus of intellectual interest to consider a supplementary game in which every player has one shot to vaccinate and in which she chooses when to obtain the vaccine as the epidemic spreads. We leave this extension for future research.

## Supporting information

S1 Data (RAR)

S1 Appendix Model and predictions. (PDF)

S2 Appendix Experimental instructions (Treatment 1)—For online publication only. (PDF)

S1 Fig Time trend. Add descriptive text after the title of the item (optional).
(EPS)

S2 Fig Time trend—Treatment 3L. (EPS)

S3 Fig Screen shot—Your decision (TIF)

S4 Fig (EPS)

## References

[1] Laxminarayan R, Mills AJ, Breman JG, Measham AR, Alleyne G, Claeson M, et al Advancement of global health: key messages from the Disease Control Priorities Project. The Lancet. 2006;367(9517):1193–1208. 10.1016/S0140-6736(06)68440-7 16616562

[2] WHO Ebola Response Team. West African Ebola epidemic after one year-slowing but not yet under control. N Engl J Med. 2015;372(6):584–7. 10.1056/NEJMc1414992 25539446 PMC4368109

[3] Van Bavel J, Baicker K, Boggio P, Capraro V, Cichocka A, Cikara M, et al. Using social and behavioural science to support COVID-19 pandemic response. Nature Human Behavior;. 10.1038/s41562-020-0884-z 32355299

[4] Kermack WO, McKendrick AG. A contribution to the mathematical theory of epidemics. Proceedings of the royal society of london Series A, Containing papers of a mathematical and physical character. 1927;115(772):700–721.

[5] Bauch CT, Earn DJ. Vaccination and the theory of games. Proceedings of the National Academy of Sciences of the United States of America. 2004;101(36):13391–13394. 10.1073/pnas.0403823101 15329411 PMC516577

[6] Katz ML, Shapiro C. Network externalities, competition, and compatibility. The American economic review. 1985;75(3):424–440.

[7] Farrell J, Saloner G. Standardization, compatibility, and innovation. The RAND Journal of Economics. 1985; p. 70–83.

[8] Geoffard PY, Philipson T. Disease eradication: private versus public vaccination. The American Economic Review. 1997;87(1):222–230.

[9] Anderson RM, May RM. Infectious diseases of humans: dynamics and control. vol. 28 Wiley Online Library; 1992.

[10] Dixit A, Olson M. Does voluntary participation undermine the Coase Theorem? Journal of Public Economics. 2000;76(3):309–335. 10.1016/S0047-2727(99)00089-4

[11] Goeree JK, Holt CA, Smith AM. An experimental examination of the volunteer’s dilemma. Games and Economic Behavior. 2017;102:303—315. 10.1016/j.geb.2017.01.002

[12] Ibuka Y, Li M, Vietri J, Chapman GB, Galvani AP. Free-riding behavior in vaccination decisions: An experimental study. PloS one. 2014;9(1):e87164 10.1371/journal.pone.0087164 24475246 PMC3901764

[13] Fischbacher U. z-Tree: Zurich toolbox for ready-made economic experiments. Experimental economics. 2007;10(2):171–178. 10.1007/s10683-006-9159-4

[14] Heal G, Kunreuther H. The vaccination game. Risk Management and Decision Processes Center Working Paper. 2005;(05-10).

[15] Galeotti A, Rogers BW. Strategic Immunization and Group Structure. American Economic Journal: Microeconomics. 2013;5(2):1–32.

[16] Chen F, Toxvaerd F. The economics of vaccination. Journal of theoretical biology. 2014;363:105–117. 10.1016/j.jtbi.2014.08.003 25111844

[17] Gersovitz M, Hammer JS. The Economical Control of Infectious Diseases. The Economic Journal. 2004;114(492):1–27. 10.1046/j.0013-0133.2003.0174.x

[18] Keser C. Voluntary contributions to a public good when partial contribution is a dominant strategy. Economics Letters. 1996;50(3):359–366. 10.1016/0165-1765(95)00769-5

[19] Sefton M, Steinberg R. Reward structures in public good experiments. Journal of Public Economics. 1996;61(2):263–287. 10.1016/0047-2727(95)01534-5

[20] Isaac RM, Walker JM. Nash as an organizing principle in the voluntary provision of public goods: Experimental evidence. Experimental Economics. 1998;1(3):191–206. 10.1023/A:1009996324622

[21] Laury SK, Walker JM, Williams AW. The voluntary provision of a pure public good with diminishing marginal returns. Public Choice. 1999;99(1):139–160. 10.1023/A:1018302432659

[22] Cason TN, Gangadharan L. Promoting cooperation in nonlinear social dilemmas through peer punishment. Experimental Economics. 2015;18(1):66–88. 10.1007/s10683-014-9393-0

[23] Ostrom E, Gardner R, Walker J. Rules, games, and common-pool resources. University of Michigan Press; 1994.

[24] Casari M, Plott CR. Decentralized management of common property resources: experiments with a centuries-old institution. Journal of Economic Behavior & Organization. 2003;51(2):217–247. 10.1016/S0167-2681(02)00098-7

[25] Diekmann A. Volunteer’s dilemma. Journal of Conflict Resolution. 1985; p. 605–610. 10.1177/0022002785029004003

[26] Goeree JK, Holt CA. An explanation of anomalous behavior in binary-choice games: Entry, voting, public goods, and the volunteers’ dilemma. Unpublished paper, University of Virginia. 2000;.

[27] Capraro V, Barcelo H. Group size effect on cooperation in one-shot social dilemmas II: Curvilinear effect. PloS one. 2015;10(7). 10.1371/journal.pone.0131419 PMC4504514 26182247

[28] Palfrey TR, Rosenthal H. Participation and the provision of discrete public goods: a strategic analysis. Journal of public Economics. 1984;24(2):171–193. 10.1016/0047-2727(84)90023-9

