# Supplementary material for: Herd immunity and a vaccination game: An experimental study
Source: PLoS One. 2020 May 14;15(5):e0232652. doi: 10.1371/journal.pone.0232652 (PMC7224512; doi:10.1371/journal.pone.0232652)
Supplement: S1 Appendix — (PDF) [file pone.0232652.s002.pdf]

S1 Appendix. Model and Predictions

We reproduce the result on π_P from [5] below and we want to highlight the concavity of the infection probability as a function of the vaccination coverage.

Proposition 1. *[[5]] Given any $P \in [0,1]$, there exists a unique π_P that is strictly decreasing and concave in P until P reaches the elimination threshold P_{crit}. Furthermore, $\pi_P = 1 - \frac{1}{R_0(1-P)}$ for any $P < P_{crit}$, and $\pi_P = 0$ for any $P \geqslant P_{crit}$.*

We now proceed to analyzing the game.

Let $\sigma_i \in [0,1]$ denote the probability that player i chooses vaccination. $\boldsymbol{\sigma} = (\sigma_1, \ldots, \sigma_n)$ denotes a mixed-strategy profile. The expected payoff for player i from randomization with σ_i can be expressed as follows:

$$EU_i(\sigma_i, \sigma_{-i}) = \frac{u(R)}{\mu} - \sigma_i C - (1-\sigma_i) d_{R_0} L \mathbb{E}[\pi_{P(\boldsymbol{\sigma})}],$$

where $\mathbb{E}[\pi_{P(\boldsymbol{\sigma})}]$ denotes the expected infection probability given the mixed-strategy profile $\boldsymbol{\sigma}$.

Definition 1. *A strategy profile $\boldsymbol{\sigma}^* = (\sigma_1^*, \ldots, \sigma_n^*) \in [0,1]^n$ is a totally mixed-strategy Nash equilibrium for the game $\mathcal{G}$ if we have for any $i \in \mathcal{N}$*

$$\sigma_i \in (0,1), \tag{3}$$

and for all $\sigma_i^* \in [0,1]$,

$$EU_i(\sigma_i^*, \sigma_{-i}^*) \geqslant EU_i(\sigma_i, \sigma_{-i}^*). \tag{4}$$

In what follows, we shall focus on the case in which $C \leqslant \pi_0 d_{R_0} L$. If $C > \pi_0 d_{R_0} L$, the only equilibrium outcome is zero vaccination coverage.

Characterization of all Nash equilibria

The following proposition characterizes the set of pure-strategy equilibrium outcomes.

Proposition 2. *For $k = 0, 1, \ldots, \nu_{crit} - 1$, $\nu = k+1$ is the pure strategy equilibrium outcome for $\frac{C}{d_{R_0} L} \in (\pi_{k+1}, \pi_k]$.*

Proof of Proposition 2. For any $i \in V(\boldsymbol{b})$, she has no incentive to deviate because $C \leqslant d_{R_0} L \pi_k$; for any $i \notin V(\boldsymbol{b})$, she has no incentive to deviate because $C > d_{R_0} L \pi_{k+1}$. $\nu < k+1$ cannot arise in pure strategy equilibrium because some $i \notin V(\boldsymbol{b})$ can always be better off by taking the vaccination. $\nu > k+1$ cannot arise in pure strategy equilibrium because some $i \in V(\boldsymbol{b})$ can always be better off by not taking the vaccination. Lastly, note that $\nu > \nu_{crit}$ cannot arise in equilibrium because the infection probability vanishes. ■

We now show a general characterization of all mixed-strategy equilibria. Let $\mathcal{M}$ be the set of players using mixed strategies, and $|\mathcal{M}| = m$. The next proposition characterizes all the mixed-strategy equilibria for $m > 1$,

Proposition 3. *Given $n > R_0$, for $m > 1$ and $\nu \leqslant \min\{\nu_{crit} - 1, n - m\}$, $\langle \nu, m \rangle$ arises as a mixed strategy equilibrium outcome for $\frac{C}{d_{R_0} L} \in (\pi_{\nu+m-1}, \pi_\nu)$ with $\boldsymbol{\sigma} = \boldsymbol{\sigma}^*$ and is uniquely determined by*

$$\frac{C}{d_{R_0} L} = \sum_{k=0}^{m-1} \pi_{\nu+k} \binom{m-1}{k} \sigma^{*k} (1 - \sigma^*)^{m-1-k}. \tag{5}$$

Proof of Proposition 3. A mixed-strategy Nash equilibrium requires that every player in $\mathcal{M}$ is indifferent between vaccination and non-vaccination, i.e.,

$$EU_i(vc, \sigma^*_{-i}) = EU_i(nv, \sigma^*_{-i}) \quad \text{for any } i \in \mathcal{M}. \tag{6}$$

It follows that

$$\frac{1}{\mu} u(R) - C = \frac{1}{\mu} u(S) - d_{R_0} L \mathbb{E}[\pi_{P(\sigma)}].$$

where $\mathbb{E}[\pi_{P(\sigma)}]$ denote the expected infection probability given $\nu = k$. Note that the additional vaccination arising from mixed-strategy follows the Poisson binomial distribution with success probabilities σ_{-i}, we obtain

$$\frac{C}{d_{R_0} L} = \sum_{k=0}^{m-1} \pi_{\nu+k} \sum_{V \in \mathcal{P}(\mathcal{M}_{-i}; k)} \prod_{j \in V} \sigma_j \prod_{l \in \mathcal{M}_{-i}/V} (1 - \sigma_l) \tag{7}$$

for any $i \in \mathcal{M}$. Consider this system of equations (characterizing the indifference conditions for the players in set $\mathcal{M}$) where $\nu \leqslant \min\{\nu_{crit} - 1, n - m\}$, we claim that for any mixed strategy equilibria with $m > 1$, the mixed-strategy profile $\boldsymbol{\sigma}$ is unique as shown by the following two lemmas.

Lemma 1. *There exists a solution to (7) for $\frac{C}{d_{R_0} L} \in (\pi_{\nu+m-1}, \pi_\nu)$ s.t. $\sigma_i = \sigma^*$ for any $i \in \mathcal{M}$.*

Proof of Lemma 1. The system (7) reduces to

$$\frac{C}{d_{R_0} L} = \sum_{k=0}^{m-1} \pi_{\nu+k} \binom{m-1}{k} \sigma^{*k} (1 - \sigma^*)^{m-1-k}. \tag{8}$$

By intermediate value theorem, there exists $\sigma^* \in (0, 1)$ such that the above equation holds. ■

Lemma 2. *(7) has at most one solution.*

Proof of Lemma 2. Define vector-valued function $H\colon [0,1]^n \to \mathbb{R}^n$ where every

component function

$$H_i := d_{R_0} r \sigma_i \sum_{k=0}^{m-1} \pi_{\nu+k} \sum_{V \in \mathcal{P}(\mathcal{M}_{-i};k)} \prod_{j \in V} \sigma_j \prod_{l \in \mathcal{M}_{-i}/V} (1-\sigma_l).$$

It is easy to check that H is continuously differentiable on $(0,1)^n$. The system of equations (7) is equivalent to $\sigma_i = H_i(\boldsymbol{\sigma})$ for all $i \in \mathcal{N}$. Suppose there exists two solutions $\boldsymbol{\sigma}^*$ and $\boldsymbol{\sigma}'$ such that $\|\boldsymbol{\sigma}^* - \boldsymbol{\sigma}'\| > 0$. By mean value inequality (Rudin, 1976), we have

$$\|\boldsymbol{\sigma}^* - \boldsymbol{\sigma}'\| = \|H(\boldsymbol{\sigma}^*) - H(\boldsymbol{\sigma}')\| \leqslant \|DH(\xi)\| \cdot \|\boldsymbol{\sigma}^* - \boldsymbol{\sigma}'\| \tag{9}$$

where $\xi \in (0,1)^n$ and $DH(\xi)$ is the Jacobian matrix evaluated at ξ. Since the row vectors of $DH(\xi)$ are linearly dependent, $DH(\xi)$ is not invertible and thus $\|DH(\xi)\| = 0$. It follows that $\|\boldsymbol{\sigma}^* - \boldsymbol{\sigma}'\| \leqslant 0$. The requires a contradiction. ■

Combining the two lemmas, we reach the conclusion that in any mixed strategy equilibrium with $m > 1$, mixing probabilities must be unique and identical across players.

Now (5) implies the best response of any $i \in \mathcal{M}$. Any $i \in V(\boldsymbol{b})$ has no incentive to deviate since her incentive constraint $C < d_{R_0} L \mathbb{E}[\pi_{k-1}]$ can be simplified using (5) as

$$\frac{C}{d_{R_0} L} < \sigma^{m-1} \pi_{\nu+m-2} + (1-\sigma)^{m-1} \pi_{\nu-1},$$

which holds under $\frac{C}{d_{R_0} L} \in (\pi_{\nu+m-1}, \pi_\nu)$. Any $i \notin V(\boldsymbol{b})$ has no incentive to deviate since her incentive constraint $C > d_{R_0} L \mathbb{E}[\pi_{P(\sigma)}]$ can be simplified using (5) as

$$\frac{C}{d_{R_0} L} > \sigma^{m-1} \pi_{\nu+m} + (1-\sigma)^{m-1} \pi_{\nu+1},$$

which again holds under $\frac{C}{d_{R_0} L} \in (\pi_{\nu+m-1}, \pi_\nu)$. This completes the proof. ■

It follows from the general proposition that there exists a unique totally mixed strategy equilibrium where $m = n$.

Corollary 1. *There exists a unique totally mixed strategy equilibrium, where $\sigma_i^* = \sigma^*$ and is implicitly defined by*

$$\frac{1}{d_{R_0} r} = \sum_{k=0}^{\nu_{crit}} \left(1 - \frac{1}{R_0} - \frac{k}{n}\right) \binom{n}{k} \sigma^{*k} (1-\sigma^*)^{n-1-k}. \tag{10}$$

Equilibrium Vaccination Coverage

Let $\mathcal{P}^*(r, R_0)$ denote the probability distribution over the vaccination coverage induced in the mixed-strategy equilibrium. The following proposition shows that an increase in the relative benefit r leads to an equilibrium distribution yielding an unambiguously higher vaccination coverage.

Proposition 4. *For any $R_0 > 1$, $\mathcal{P}^*(r, R_0)$ first-order stochastically dominates (FOSD) $\mathcal{P}^*(r', R_0)$ if and only if $r > r'$.*

Proof of Proposition 4. Let ν and ν' be the equilibrium number of vaccinated people given the relative benefit r and r' respectively. For future use, denote $F_\nu(x)$ as the CDF of a random variable ν. We start by working directly with σ as shown in the following lemma.

Lemma 3. *ν FOSD ν' if and only if $\sigma > \sigma'$.*

Proof of Lemma 3. ν FOSD ν' if and only if $F_\nu(x) \leqslant F_{\nu'}(x)$ for any $x \in \{1, \ldots, n\}$. Since both ν and ν' follows binomial distribution with n trials, we remain to show $\frac{dF_\nu(x)}{d\sigma} \leqslant 0$. Now consider for any x, the derivative

$$\begin{aligned}\frac{dF_\nu(x)}{d\sigma} &= \sum_{k=1}^{x} k\binom{n}{k}\sigma^{k-1}(1-\sigma)^{n-k} - \sum_{k=0}^{x}(n-k)\binom{n}{k}\sigma^{k}(1-\sigma)^{n-k-1} \\ &= n\left(\sum_{k=1}^{x}\binom{n-1}{k-1}\sigma^{k-1}(1-\sigma)^{n-k} - \sum_{k=0}^{x}\binom{n-1}{k}\sigma^{k}(1-\sigma)^{n-k-1}\right) \\ &= n\left(F_{\tilde{\nu}}(x-1) - F_{\tilde{\nu}}(x)\right) \leqslant 0\end{aligned}$$

where $\tilde{\nu} \sim Bin(n-1, \sigma)$. ■

It remains to show that σ^* is monotonically increasing in r. By implicit function theorem, taking partial derivative of the vaccine uptake likelihood $\sigma^*(r, R_0)$ with respect to r gives us

$$\frac{\partial\sigma^*}{\partial r} = 1/\left[d_{R_0} r^2 \sum_{k=0}^{v_{crit}} ((n-1)\sigma^* - k)\left(1 - \frac{1}{R_0} - \frac{k}{n}\right)\binom{n}{k}\sigma^{*k-1}(1-\sigma^*)^{n-2-k}\right],$$

which is positive for any $r > \frac{R_0}{(R_0-1)d_{R_0}}$.

Therefore, ν FOSD ν' and equivalently, $P(r, R_0)$ FOSD $P(r', R_0)$ if and only if $r > r'$. ■

The stochastic dominance presented in Proposition 4 implies that $Pr^*(P \geqslant P_{crit})$, the equilibrium probability for the society to achieve the vaccination coverage needed to obtain herd immunity, is monotonically increasing in r and converges to 1 as r goes to infinity. By nature of mixed-strategy equilibria, it is impossible to obtain $Pr^*(P \geqslant P_{crit}) = 1$ and obliterate an epidemic. However, the society can still

approximate the complete immunity via voluntary vaccination. The following corollary summarizes this discussion.

Corollary 2. $\Pr^*(P \geqslant P_{crit}) \to 1$ *as* $r \to \infty$.

Proof of Corollary 2. By Proposition 4, $\Pr^*(P \geqslant P_{crit}) = 1 - F_\nu(\nu_{crit})$ is monotonically increasing in r. Furthermore, as $r \to \infty$, $\sigma^* \to 1$, and $F_\nu(\nu_{crit}) \to 0$. ■

We now investigate the role of concavity of the long-run infection probability in facilitating the elimination of an epidemic. Let $\mathcal{P}^L(r, R_0)$ be the equilibrium vaccination coverage in the linearized environment with $\pi^L_P = 1 - \frac{1}{R_0} - P$ for any $P < P_{crit}$, and $\pi^L_P = 0$ for any $P \geqslant P_{crit}$. Then we have the following result.

Proposition 5. *For any* $r > \frac{R_0}{(R_0-1)d_{R_0}}$ *and* $R_0 > 1$, $\mathcal{P}^*(r, R_0)$ *FOSD* $\mathcal{P}^L(r, R_0)$.

Proof of Proposition 5. By Proposition 3, the totally mixed strategy equilibrium in the linearized environment $\sigma^L_i = \sigma^L$ is implicitly defined by

$$\frac{1}{d_{R_0} r} = \sum_{k=0}^{\nu_{crit}} \left(1 - \frac{1}{R_0} - \frac{k}{n}\right) \binom{n-1}{k} {\sigma^L}^k (1 - \sigma^L)^{n-1-k}. \tag{11}$$

By Pascal's rule, $\binom{n-1}{k} \leqslant \binom{n}{k}$ for any $k < n$ and thus $\sigma^L \leqslant \sigma^*$. By lemma 3, $P^*(r, R_0)$ FOSD $P^L(r, R_0)$. ■

We now address a comparative static question regarding R_0. That is, are the players more likely to reach immunity if they are faced with a more threatening epidemic? An exogenous increase in the reproduction ratio R_0 has two competing effects on how easily the society can achieve herd immunity via voluntary vaccination. On the one hand, it raises the long-run probability of infection π_P, meaning that individuals in the mixed-strategy Nash equilibrium are more likely to vaccinate. On the other hand, a higher R_0 also increases the critical level needed for herd immunity P_{crit}. Figure 1 shows that the first effect dominates the second effect so that it is easier to achieve herd immunity when R_0 is higher. This result is summarized in the following proposition.

Proposition 6. *For any* $r \in (\frac{R_0}{(R_0-1)d_{R_0}}, +\infty)$, $\mathcal{P}^*(r, R_0)$ *FOSD* $\mathcal{P}^*(r, R_0')$ *if and only if* $R_0 > R_0'$.

Proof of Proposition 6. By implicit function theorem, taking partial derivative of the vaccine uptake likelihood $\sigma^*(r, R_0)$ with respect to R_0 gives us

$$\frac{\partial \sigma^*}{\partial R_0} = \frac{\sum_{k=0}^{v_{crit}} {R_0}^{-2} \binom{n}{k} {\sigma^*}^k (1 - \sigma^*)^{n-1-k} + \frac{\dot{d}_{R_0}}{d^2_{R_0} r}}{\sum_{k=0}^{v_{crit}} \left((n-1)\sigma^* - k\right) \left(1 - \frac{1}{R_0} - \frac{k}{n}\right) \binom{n}{k} {\sigma^*}^{k-1} (1 - \sigma^*)^{n-2-k}}, \tag{12}$$

which is positive for $r > \frac{R_0}{(R_0-1)d_{R_0}}$. By lemma 3, $P^*(r, R_0)$ FOSD $P^*(r, R_0')$ if and only if $R_0 > R_0'$. ■

This result implies that a more contagious disease is unambiguously easier to deal with. A higher R_0 encourages people to get vaccinated voluntarily. Hence, epidemics like Ebola, with substantially low R_0, are particularly difficult to control based on voluntary vaccination.
