# Supplementary material for: Herd immunity and a vaccination game: An experimental study
Source: PLoS One. 2020 May 14;15(5):e0232652. doi: 10.1371/journal.pone.0232652 (PMC7224512; doi:10.1371/journal.pone.0232652)
Supplement: S2 Appendix — (PDF) [file pone.0232652.s003.pdf]

S2 Appendix. Experimental Instructions (Treatment 1) – For Online Publication Only

INSTRUCTION

Welcome to the experiment. This experiment studies decision making in groups of eight individuals. In the following two hours or less, you will participate in 20 rounds of decision making. Please read the instructions below carefully; the cash payment that you will receive at the end of the experiment depends on how well you make your decisions according to these instructions. If you have a question at any point, please raise your hand and wait for one of us to come over. We will then privately answer your question. We ask that you turn off your mobile phone and any other electronic devices. Communication of any kind with the other participants is not allowed.

Your Group

In each and every round, you will be *randomly* matched with seven other participants to form a group of eight. You will not be told the identity of the participants with whom you are matched, nor will these participants be told your identity—even after the end of the experiment. In each group, eight members are required to make decisions that will affect their earnings in the round. Participants will be randomly rematched after each round to form new groups.

Your Decision

Screen_Sample_T1-eps-converted-to.pdf

Figure 7. Screen Shot – Your Decision

There are eight individuals (including yourself) in your group. Suppose that these eight individuals are living in a village. Initially, every individual in the village begins with the same status of green. Figure 7 illustrates this situation. The green circle at the center represents yourself, and the other seven black circles represent other individuals in the village. The red circle carries the source of redness from which you want to protect yourself.

The only way for you to protect yourself from the redness is to purchase a shield. With the shield, you will be immune to the redness and stay green; Without the shield, you will either turn red or remain green. Without the shield, the chance of your turning ted depends on how many other

individuals in the village have the shield. Table 5 below (or the table at the top-right corner of Figure 7) presents the probability of turning red. Note that, when four individuals or more in your village choose to buy the shield, everyone will stay green regardless of whether he/she buys the shield or not.

If you choose "No Shield"	
# of others having the shield	Chance of your turning red
0	50.0%
1	42.9%
2	33.3%
3	20.0%
4	0.00%
5	0.00%
6	0.00%
7	0.00%

Table 5. Probability of your turning red

Each individual independently and simultaneously decides whether to buy the shield or not. You will be prompted to make your decision by clicking one of the two buttons, "Shield" and "No Shield," presented at the bottom of your screen. Once you click one of the buttons, your decision in the round is completed.

Your Earnings

Your earning in each round depends on: 1) whether or not you buy the shield; and 2) your status. Table 6 below summarizes your earnings, in which the earnings are expressed in terms of the experimental currency units (ECU).

Your decision	Shield	No Shield	
Your status	Green	Green	Red
Your earning	75	80	72

Table 6. Your Earnings

Information Feedback

At the end of each round, the computer will provide a summary for the round: your decision (Shield or No Shield), your final status (Green or Red), your earnings, and the number of people having the shield in your group.

Your Cash Payment

The experimenter randomly selects 1 round to calculate your cash payment. Each round has an equal chance of being selected (so it is in your best interest to consider each round seriously.) Your final cash payment will be your earnings (1 ECU = 1 HKD) in the selected round, plus a HK$30 show-up fee.

Practice Rounds

To ensure your understanding of the instructions, we will provide you with a quiz and a practice round. We will go through the quiz after you answer it on your own. You will then participate in 1 practice round. The practice round is part of the instructions that are not relevant to your cash payment; its objective is to familiarize you with the computer interface and the flow of the decisions in each round. Once the practice round is over, the computer will tell you "The official rounds begin now!"

Administration

Your decisions, as well as your monetary payment, will be kept confidential. Remember that you must make your decisions entirely on your own; please do not discuss your decisions with any of the other participants. Upon finishing the experiment, you will receive your cash payment. You will be asked to sign your name to acknowledge your receipt of the payment (which will not be used for tax purposes). You are then free to leave. If you have any questions, please raise your hand now. We will answer your questions individually. If there are no questions, we will proceed to the quiz.

Quiz

1. True or False: I will be matched with the same seven other players in all 20 rounds.
2. True or False: I will turn red for sure if I do not have the shield.
3. Suppose that you decide to buy the shield. Calculate your earnings. __________
4. Suppose that you decide not to buy the shield. It turns out that there are five other individuals in your group who buy the shield. Calculate your earnings. __________
5. Suppose that you decide not to buy the shield. It turns out that there are two other individuals in your group who buy the shield. What is the chance of your turning red? __________
