# Supplementary figures and images for: Herd immunity and a vaccination game: An experimental study
Source: PLoS One. 2020 May 14;15(5):e0232652. doi: 10.1371/journal.pone.0232652 (PMC7224512; doi:10.1371/journal.pone.0232652)

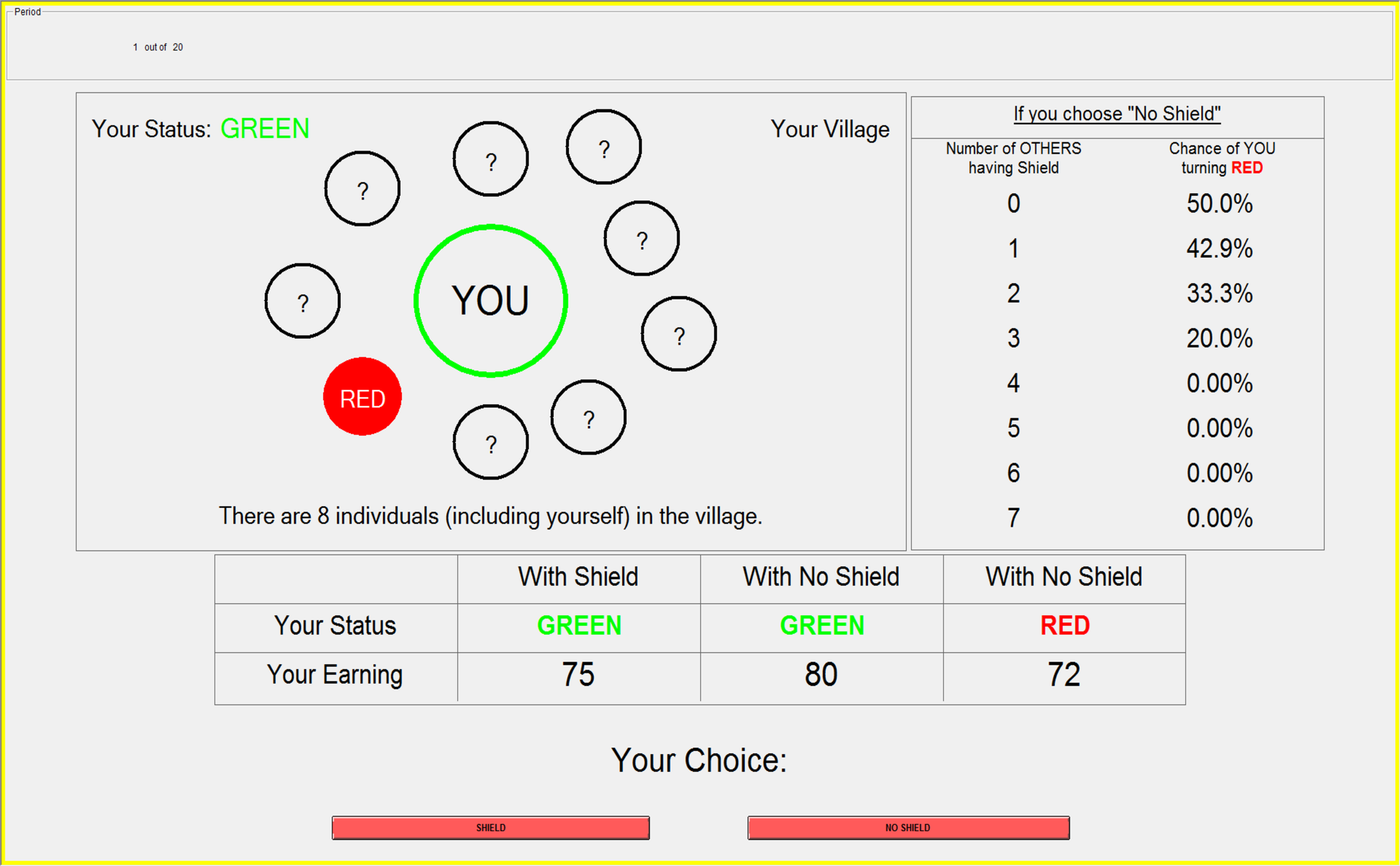

Supplement: S3 Fig — (TIF) [file pone.0232652.s006.tif]
